# Analysis of image-based sow activity patterns reveals several associations with piglet survival and early growth

**DOI:** 10.3389/fvets.2022.1051284

**Published:** 2023-01-09

**Authors:** Océane Girardie, Mathieu Bonneau, Yvon Billon, Jean Bailly, Ingrid David, Laurianne Canario

**Affiliations:** ^1^UMR1388 GenPhySE, INRAE, Université de Toulouse, INPT, Castanet-Tolosan, France; ^2^UR0143 ASSET, INRAE, Petit-Bourg, France; ^3^UE GenESI, INRAE, Surgères, France

**Keywords:** computer vision, convolutional neural network, clustering, longitudinal analysis, piglet survival, piglet growth, sow activity pattern

## Abstract

An activity pattern describes variations in activities over time. The objectives of this study are to automatically predict sow activity from computer vision over 11 days peripartum and estimate how sow behavior influences piglet's performance during early lactation. The analysis of video images used the convolutional neural network (CNN) YOLO for sow detection and posture classification of 21 Large White and 22 Meishan primiparous sows housed in individual farrowing pens. A longitudinal analysis and a clustering method were combined to identify groups of sows with a similar activity pattern. Traits under study are as follows: (i) the distribution of time spent daily in different postures and (ii) different activities while standing. Six postures were included along with three classes of standing activities, i.e., eating, drinking, and other, which can be in motion or not and root-pawing or not. They correspond to a postural budget and a standing-activity budget. Groups of sows with similar changes in their budget over the period (D-3 to D-1; D0 and D1–D7) were identified with the k-means clustering method. Next, behavioral traits (time spent daily in each posture, frequency of postural changes) were used as explanatory variables in the Cox proportional hazards model for survival and in the linear model for growth. Piglet survival was influenced by sow behavior on D-1 and during the period D1–D7. Piglets born from sows that were standing and doing an activity other than drinking and eating on D-1 had a 26% lower risk of dying than other piglets. Those born from sows that changed posture more frequently on D1–D7 had a 44% lower risk of dying. The number of postural changes, which illustrate sow restlessness, influenced piglet growth in the three periods. The average daily gain of piglets born from sows that were more restless on D1–D7 and that changed posture more frequently to hide their udder on D0 decreased by 22 and 45 g/d, respectively. Conversely, those born from sows that changed posture more frequently to hide their udder during the period of D1–D7 grew faster (+71 g/d) than the other piglets. Sow restlessness at different time periods influenced piglet performance.

## 1. Introduction

Improving piglet performance in housing conditions where sows have greater freedom of movement is a major economic, ethical, and societal concern. One way to address this need for indoor production is to promote the housing of lactating sows in individual pens. This design allows sows to interact more with their piglets as well as to avoid their demands for more milk (1–3). Piglet survival can be impaired, either due to crushing by the sow (4–6) or starvation [7–16% (6)], both of which tend to increase in individual pens (7, 8). This is particularly true for sows that are less maternal and that lie down abruptly (8–10) or that suffer from a lactation problem (11).

Sow behavior varies greatly from day to day in the peripartum period (12–14). Therefore, the analysis of the sow activity pattern, which refers to the evolution of behavior with time, may be essential for addressing the issue of piglet performance. The activity pattern of sows is lowly described in the literature due to the lack of monitoring tools. It has been mainly assessed by direct observations, within short time windows and with a limited number of animals (12, 15, 16). After a nest-building activity phase in the 24–48 h before farrowing, sows calm down and remain lying most of the time during the farrowing process (17, 18). Thereafter, they gradually resume activity (12, 19), which is then punctuated by nursing bouts (3). Some of the sow body movements, from standing to lying and from sternal lying to lateral lying, increase the risk of piglet crushing (20). This occurs more frequently in sows that move to prevent the piglets from suckling more when they change posture to hide their udder, often under the belly (21).

Sow behavior can also vary considerably among individuals, and genetic background influences behavior (22), which is illustrated by differences between breeds. Large White (LW) and Meishan (MS) sows markedly differ in reactivity, the former responding to disturbances in the environment and the latter remaining placid under the vast majority of situations (23–25). Indeed, selective breeding has endowed LW sows with good maternal abilities oriented toward the nursing of a large number of fast-growing piglets, and they produce more milk than MS sows (26, 27). The MS sows have good maternal abilities as well, as seen by the high survival rate of their piglets. They also have a more pronounced exploratory behavior and they interact more with their piglets than LW sows (25, 27). However, they do not seem to markedly differ from LW sows in their postural budget (27, 28), i.e., the amount of time spent in the different postures.

An increasing number of research teams rely on artificial intelligence methods for image analysis of animal behavior. Methods based on the Convolutional Neural Networks (CNN) are being developed to estimate the postures and activities of sows in individual pens (29, 30) and will make it possible to access longitudinal information (31). This approach is adequate for studying large populations. The objective of the current study was to apply CNNs on video records of 43 sows over a continuous period of 11 days peripartum to identify sow behavioral traits that have an influence on piglet survival and growth. We used CNNs to predict the postural activity and standing activity of sows. From predictions, we investigated time budgets, i.e., the amount of time spent in the different postures and specific standing activities. Then, we identified groups of sows that differed in their activity patterns and estimated the influence of sow behavior on piglet performance.

## 2. Materials and methods

### 2.1. Experimental design

**Figure 1:**
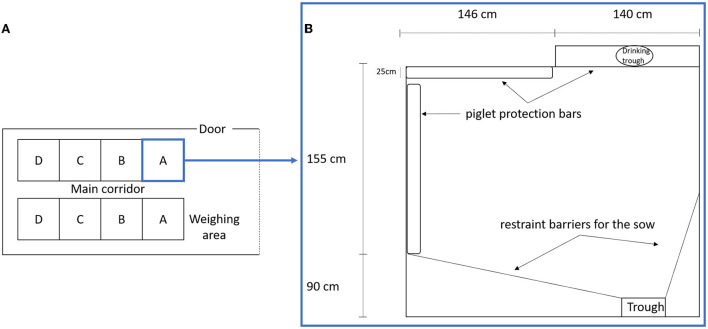
**(A)** Scheme of farrowing unit room, A, B, C, and D, represents the pen position in relation to the principal door and weighing area. **(B)** Scheme of a farrowing pen.

This study included 21 LW and 22 MS primiparous sows raised at the INRAE GenESI experimental farm of Le Magneraud (10.15454/1.5572415481185847E12). The animals were reared in five successive batches between November 2010 and January 2011. The farrowing unit contained two rooms (Figure 1A) which housed batches at 3 weeks interval. The sows entered the farrowing unit ~7 days before the expected farrowing date. The LW and MS sows were placed alternately in adjacent pens, which were 286 cm × 255 cm and contained protective bars along the walls as well as a creep area with a heat lamp (Figure 1B). Restraining bars were in open mode and hardly ever used on a very short period for sows that savaged piglet(s) (*N* = 3). The floor was covered with a thin bed of straw, which was changed every morning, the quantity of which was adjusted around the time of farrowing according to its use by the sow. The straw had a positive impact on sow activity (1), especially to perform nest building. Sows were fed two times a day at 8 a.m. and 4 p.m. The feeding trough and the drinking trough were on opposite sides of the pen. From the day of entrance in the farrowing unit, sows were daily accustomed to human presence and interventions for routine and experimental procedures.

The animals were part of a crossbreeding design. Each sow was inseminated with mixed semen of LW and MS boars to produce purebred and crossbred piglets in the same litter. As a consequence, four piglet genetic types were produced (dam/sire): LW purebred (LW/LW), MS purebred (MS/MS), LW crossbred (LW/MS), and MS crossbred (MS/LW). Immediately at birth, the piglets were individually caught and carried out of the pen to be dried and weighed in the central corridor of the farrowing unit (Figure 1A). The rooms were isolated from the central corridor by a plastic curtain. Piglets were weighed again on D1 (24 h after birth), D3, and D7. The total number of piglets born was counted at birth. Each morning, animal keepers recorded the number of dead piglets and identified the causes of death, distinguishing stillbirth, crushing by the sow, starvation, and other causes. All sows were filmed on 24-h day, from D-3 until D7, using a 2D digital camera fixed on the ceiling of each pen to have a view that precisely covered the whole surface of the pen.

### 2.2. Behavioral measurements and annotation

Behavioral traits that described sow postural activity and sow standing activity were derived from the ethogram presented in Table 1. Six different postures were considered: Standing (ST), Sitting (SI), Kneeling (K), Lateral Lying (LL), Lateral Lying with udder exposed (LLU), and Sternal Lying (SL). While standing, three activities where identified: drinking, eating, and other, which can be in motion or not and rooting or not.

**Table 1:** Ethogram of sow postural activity and standing activity.

**Posture**	
Sternal lying	Lying ventrally with udder unexposed
Lateral lying	Lying laterally with udder unexposed
Lateral lying with udder exposed	Lying laterally with udder visible
Sitting	Standing on its hindquarters with the front legs not bent
Kneeling	The knees of the front legs touch the ground and the back legs are straight
Standing	The sow rests on its four right legs
**Standing activity**	
Eat	The sow has her head in the feeder
Drink	The sow has her head in the drinking trough
Something other	The sow is standing without eating or drinking, and is in motion or not and pawing, rooting or not

### 2.3. CNNs' use for sow behavior detection

We used two CNNs to obtain continuous information on the postural activity and standing activity of each sow. First, we used a Yolo-v2 object detection CNN to detect the sow on the image and predict her posture. We trained Yolo-v2 (32) with six classes for the six postures previously defined. Yolo was combined with resNet50 (33) for feature extraction. To train Yolo, we used 8,400 images randomly selected from all the videos and manually labeled according to the posture. Yolo was trained using a classic stochastic gradient decent algorithm with a momentum of 0.9 for 250 epochs with a mini-batch, including 16 images. To validate the posture prediction from Yolo, 25,830 images were selected randomly, manually annotated, and compared to Yolo prediction. Second, we fitted another Yolo-v2 CNN to detect the head of the sow. Only 860 images from the previous training database were manually annotated for training. Validation of the prediction was performed on thousands of images through visual assessment, and no error was observed in the head detection.

For each image, the two Yolo models provided three informative items: (i) a bounding box around the sow to determine the center of gravity of her body, (ii) a bounding box around her head to determine the center of gravity of her head, and (iii) the posture of the sow. The two centers of gravity for the head and the body were initially in pixels. An image registration technique was used to convert the location into spatial coordinates (34). It was then possible to calculate the distance between the sow head and the trough or the feeder to detect drinking and eating activities, respectively. While standing, a sow not drinking or eating was classified as doing something other.

The MATLAB software (R2020b) was used to train the two CNNs and run the image analysis. After validation, the two CNNs were applied to create a longitudinal database describing the changes in postures and standing activity of sows with one observation every 5 s.

### 2.4. Statistical methods

The statistical analyses were carried out using R software [version R 4.1.0 (35)]. The database contained more than 8 million observations, with one observation every 5 s for each of the 11 days and for each of the 43 sows. Postures lasting only one observation were considered false predictions and were replaced by the posture detected at the previous observation. We extracted five behavioral traits from the image analysis computed on a daily basis. They were the proportion of time spent in each posture and the proportion of time spent in each activity while standing, which corresponded to the postural budget and standing-activity budget, respectively. We also computed the total number of postural changes per day (PCAll) to depict sow restlessness. We calculated the number of postural changes at risk of crushing for the piglet (PCRiskCrush). They corresponded to changes from standing to lying (LL, LLU, and SL) and from sternal lying to lateral lying (udder exposed or not) (9, 10, 20, 36–38). Finally, we computed the daily number of postural changes usable to end a nursing bout (PCStopNurse; i.e., to hide the udder) that corresponded to changes from lateral lying with the udder exposed to any other posture (3, 39). These five variables were studied over three different periods: before farrowing (bf) from D-3 to D-1 the day of farrowing D0 (df), defined as the 24-h after the birth of the first piglet, and the period after farrowing (af) from D1 to D7.

#### 2.4.1. Cluster analysis

To identify the sow activity patterns, we applied a clustering analysis on the postural budget and the standing-activity budget in three periods. To take into account the compositional aspect of budget data, i.e., the sum of proportions equal to 100%, an isometric log-ratio (ILR) transformation was first applied to the two budgets (40). Then, k-means clustering approaches were applied to identify different posture patterns and different standing-activity patterns. The klm3d package was used (41) to take into account the longitudinal aspect of the data in the before-farrowing and after-farrowing periods, as those periods included several days each. The classic k-means was used to analyze clusters on the day of farrowing. The optimal number of clusters was defined based on the Elbow method (42). As per period *k* (*k* = bf, df or af), the resulting postural clusters were labeled *A*_*k*_, *B*_*k*_… and the activity clusters were labeled *A*_*ak*_, *B*_*ak*_… .

#### 2.4.2. Survival analysis

In the analysis of piglet survival, we defined the deaths of live-born piglets as events. When piglets were alive at weaning, the data were censored. We used a Kaplan Meier (KM) approach to estimate survival probabilities for each piglet genetic type. Then, the Cox proportional hazard models were enabled to estimate the influence of sow behavior on piglet survival, following the methodology described hereafter. The R Survival package was used (43).

In the first step, each of the five behavioral items (postural pattern, standing-activity pattern, PCall, PCRiskCrush, and PCStopNurse) were considered one by one as an explanatory variable and for this, analyses were run separately in each of the three periods. The aim of this first step was to determine which variables to be included in the model with which coding, i.e., continuous or categorized. Only behavioral traits that made sense per period were tested. Thus, on D-3 to D-1, only the postural pattern, the standing-activity pattern, and PCAll were tested. PCStopNurse was not tested on D0. The best coding for PCAll, PCStopNurse, and PCRiskCrush was chosen using the Akaike information criterion (AIC). Cox models included each variable as continuous or categorized into three classes. For each continuous variable and period, the grouping in classes was based on the quantiles of distribution: class Inferior (I, values ≤ 33th percentile), class Moderate (M, 33 < values ≤ 66th percentile), and class Superior (S, values > 66th). First, we tested in the Cox models that the effects of the postural pattern and the standing-activity pattern using the clusters are previously identified as explanatory variables in each period. If the effect of the clusters was significant (*p* < 0.05), then we tested the effect of the raw behavioral traits that mainly explained the difference between the clusters. The choice of cluster or raw behavioral traits (i.e., time spent in specific postures or specific standing activities) is based on AIC.

In the second step, all factors with a *p* lower than 0.05 in the first step of the analysis were included in three saturated Cox models fitted separately in each period. Variables were then selected with a step-by-step descending procedure using the likelihood ratio test as a selection criterion to obtain the three reduced Cox models.

In the third step, the remaining significant factors (*p* < 0.05) were included in the global model that included factors from the three periods together. Again, variables were selected with a step-by-step descending procedure to obtain the final model.

For all analyses, the fixed effects of piglet genetic type (four classes), litter size (three levels: 12–14; 15–16 and 17–21 piglets born alive), and pen (four levels A–D; defined by distance from the central corridor from nearest to farthest, Figure 1A) were included in the models in addition to the sow behavioral trait(s). The proportional hazards assumption was verified using the Schoenfeld residuals for each variable included in the Cox models.

#### 2.4.3. Growth analysis

We used linear models for the analysis of piglet growth. To estimate the influence of sow behavior on the average daily gain (ADG) of the piglet from D0 to D7, we included the postural pattern, the standing-activity pattern, PCAll, and PCStopNurse as explanatory variables. The same approach in three steps as described above for analyzing piglet survival was applied to select behavioral traits with a significant effect on piglet growth.

## 3. Results

### 3.1. Image-based predictions and behavior database

**Figure 2:**
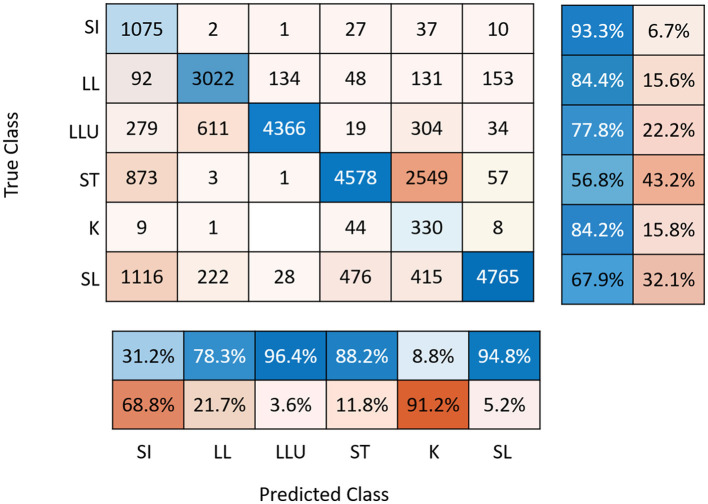
Confusion matrix associated with YOLO prediction with sensitivity **(right panel)** and precision **(below panel)**. SI, sitting; LL, lateral lying; LLU, lateral lying with udder exposed; ST, standing; K, kneeling; SL, sternal lying.

For some days and sows, video recordings were not analyzed due to video recording problems. The percentage of days with missing observations was low (7.5% of the global database). The precision of the prediction of the CNNs was 31% for posture SI, 88% for ST, 9% for K, 95% for SL, 96% for LLU, and 78% for LL (Figure 2) and did not differ between breeds (result not shown). As they were highly confounded, the K and S postures were grouped together for the analyses. Descriptive statistics for daily postural changes are given in Table 2. The number of PCAll per day was on average 266 ± 171. The number of PCStopNurse and PCRiskCrush per day was 67 ± 56 and 51 ± 35 on average. Values for each type of postural change did not differ significantly between days. However, the mean values for PCAll, PCRiskCrush, and PCStopNurse were highest on D-1.

**Table 2:** Mean number of postural changes (±SD) for each category of posture change.

**Days**	**PCAll**	**PCRiskCrush**	**PCStopNurse**
−3	220 ± 127	45 ± 26	54 ± 40
−2	297 ± 167	52 ± 26	76 ± 53
−1	470 ± 215	106 ± 54	82 ± 39
0	282 ± 183	53 ± 44	75 ± 56
1	247 ± 168	42 ± 31	65 ± 62
2	257 ± 199	41 ± 28	69 ± 80
3	219 ± 150	41 ± 24	61 ± 57
4	241 ± 181	39 ± 23	70 ± 75
5	236 ± 142	44 ± 26	67 ± 55
6	266 ± 154	55 ± 32	71 ± 53
7	253 ± 142	52 ± 32	65 ± 48

PCAll is the total number of postural changes, PCRiskCrush is the number of changes from standing to lying and from sternal lying to lateral lying, and PCStopNurse is the number of changes from lying with udder exposed to any other posture.

On average, over the whole period, sows spent 21% of their standing time drinking and 27% eating. During the D-3 to D-1 period, the percentage of time spent drinking increased from 19 to 26%. The average percentage of time spent eating decreased from 27% on D-3 to 21% on D-1. On the day of farrowing, sows spent 24% of their standing time drinking and 22% eating. During the D1–D7 period, the overall trend was an increase in the percentage of time spent eating when standing from 24% at D1 to 30% at D7.

On average over the whole period, sows spent 12% standing, 3% sitting, and 85% lying. On the D-3 to D-1 period, the percentage of time spent standing increased from 15% to 29%. Sows spent 77% of time lying (29% lateral lying, 36% lateral lying with the udder exposed, and 12% sternal lying). On the day of farrowing, sows spent more time lying (92%) and the time spent standing decreased from 29% to 8%. On average over the D1–D7 period, sows spent 89% lying (90% on D1, 89% on D7). On this period, we did not observe a significant trend in the postural pattern, and sows spent 11% of their time standing and most of the time spent lying laterally with the udder exposed (73%).

### 3.2. Clustering

**Figure 3:**
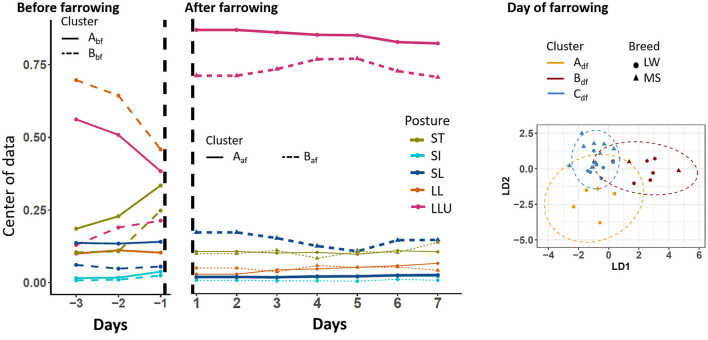
Longitudinal changes in sow postural activity according to the clusters that were identified before, after, and on the day of farrowing. SI, sitting; LL, lateral lying; SL, sternal lying; LLU, lateral lying udder exposed; ST, standing; LW, Large-White; MS, Meishan. The centers of data are shown in the periods before and after farrowing because longitudinal analyses were applied. The two first components of the linear discriminant analysis are shown on the day of farrowing.

To perform the analysis on a given period, only sows that had a complete period of observation were kept. We used 28, 30, and 33 sows on the three periods D-3 to D-1, D0, and D1–D7, respectively. The centers of the postural budget for each identified cluster are presented in Figure 3. During the D-3 to D-1 period, two postural clusters were highlighted (A_bf_ and B_bf_, respectively). They differed mainly by time spent standing and the preferred posture while lying: lateral lying for sows of cluster B_bf_, and lateral lying with the udder exposed for sows of cluster A_bf_. These differences in behavior reflected the differences between breeds as all sows of cluster A_bf_ were LW (17 animals), and all sows of cluster B_bf_ were MS (11 animals). On D0, three postural clusters were identified and noted A_df_, B_df_, and C_df_. Sows of cluster A_df_ (five animals, all LW) spent less time lying (91%) than sows of the two other clusters (93 and 94.5% for cluster B_df_ and C_df_, respectively). The two last clusters also differed in the preferred posture while lying. Sows in cluster B_df_ (2 MS, 6 LW) and C_df_ (9 MS, 8 LW) spent, respectively, 70% and 95% of the time with the udder exposed while they were lying. During the D1–D7 period, two postural clusters were identified (noted A_af_ and B_af_). Sows of the two clusters differed in the percentage of time spent lying with the udder exposed: on average 91% for sows of cluster A_af_ (13 MS, 9 LW) and 80% for sows of cluster B_af_ (1 MS, 10 LW).

**Figure 4:**
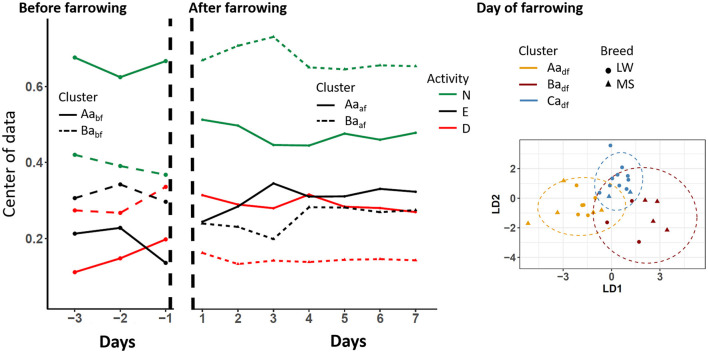
Longitudinal changes in sow standing activity according to the clusters that were identified before, after, and on the day of farrowing. D, drinking; E, eating; N, something other; LW, Large-White, MS, Meishan. The centers of data are shown in the periods before and after farrowing because the longitudinal analyses were applied. The two first components of the linear discriminant analysis are shown on the day of farrowing.

The standing-activity pattern over days and according to clusters is shown in Figure 4. Before farrowing, two activity clusters, Aa_bf_ (9 MS, 5 LW) and Ba_bf_ (1 MS, 11 LW), that differed according to time spent doing something other were identified (~66% and ~40% for cluster Aa_bf_ and Ba_bf_, respectively). It should be noted that this difference was larger on D-1. On D0, three standing-activity clusters were identified (Aa_df_, Ba_df_, and Ca_df_). The clusters Ca_af_ (12 animals, 10 LW) and Aa_af_ (5 MS, 6 LW) differed by the percentages of time spent doing something other (42% and 67% of time spent standing, respectively) and drinking (30% and 10% for Ca_df_ and Aa_df_, respectively). Conversely, sows in cluster Ba_df_ (4 MS and 3 LW) spent a very short percentage of time eating (5%) compared to sows from the other clusters (≃25%). During the D1–D7 period, two clusters were identified: Aa_af_ (5 MS, 18 LW) and Ba_af_ (10 MS, 1 LW). The main difference between groups was the percentage of time spent doing something other, which was 70% on average in cluster Ba_af_ and 46% in cluster Aa_paf_.

### 3.3. Piglet survival

**Figure 5:**
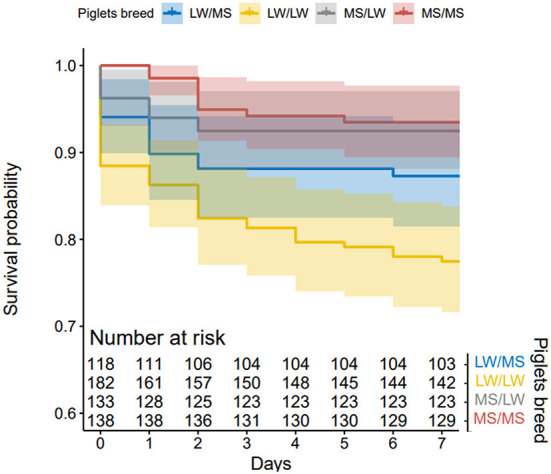
Kaplan-Meier curves of piglet mortality after farrowing for the four piglet genetic types (dam breed/sire breed).

In total, 322 piglets were born and raised by LW sows (123 LW/MS and 199 LW/LW) and 279 by MS sows (139 MS/LW and 140 MS/MS). The average litter size was 14.9 (±0.7) for LW sows and 12.7 (±0.7) for MS sows. Piglet survival in lactation was 86.8% on average, 79.9% in LW sows and 93.5% in MS sows. Results from the KM method indicated that piglet survival on D1–D7 was higher in pure MS piglets (D7 survival probability = 0.94) than in pure LW piglets (D7 survival probability = 0.78, *p* < 0.001). Crossbred piglets raised by MS sows had a similar survival to that of pure MS piglets (D7 survival probability = 0.92). In LW sows, survival was lower in pure piglets as compared to their crossbred counterparts (D7 survival probability = 0.87, *p* = 0.006, Figure 5).

**Figure 6:**
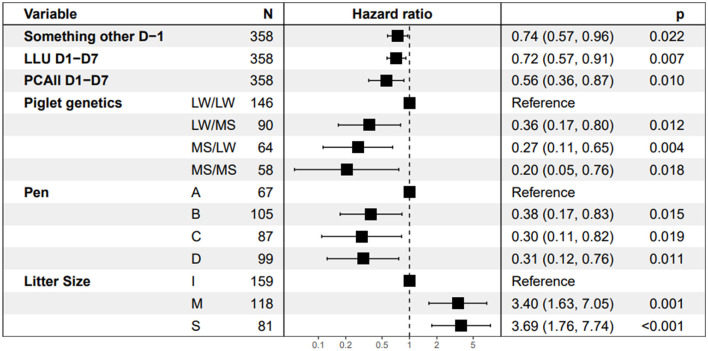
Estimates of Hazard ratios from the Cox model. “Something other D-1” is the time spent doing something other while standing, LLU D1–D7 is the time spent lying with the udder exposed after farrowing and PCAll D1–D7 is the average number of postural changes per day after farrowing divided by 100. The abbreviation for piglet genetic types is dam breed/sire breed. The four pens A, B, C, and D correspond to the location from the maternity entrance, A is the nearest, and D the farthest. For Litter Size, I represents the inferior quartile and S the superior quartile.

The behavioral traits with a significant effect on piglet survival in the final Cox model are shown in Figure 6. The three behavioral explanatory variables retained in the final model were time spent doing something other while standing on D-1, PCAll, and time spent with the udder exposed on D1–D7. Piglets from sows that spent more time lying with the udder exposed more time doing any activity other than standing or drinking while standing on D-1 and that were more restless had a lower instantaneous risk of dying than piglets raised by the other sows (HR = 0.72, 0.74, and 0.56, respectively). Piglet survival increased with restlessness on D1–D7. An increase of 100 postural changes per day led to a significant reduction in the instantaneous risk of dying by 44% based on a hazard ratio of 0.56 [95% CI (0.36, 0.87), *p* = 0.010). The time the udder was exposed on D1–D7 was also favorable to piglet survival. The instantaneous risk of dying decreased by 28% [HR = 0.72, 95% CI (0.57, 0.91), *p* = 0.007] with each supplementary hour per day spent with udder exposed on D1–D7. The longer the time spent by the sow doing something other while standing on D-1 (in hour), the higher the piglet survival was [HR = 0.74; 95%CI (0.57, 0.96), *p* = 0.022]. The instantaneous risk of dying was higher in larger litters [class S vs. I, HR = 3.69] and decreased with the distance of the sow from the central corridor in the room [near vs. far, HR = 0.31, *p* = 0.011]. Pure LW piglets had a higher risk of dying than pure MS or crossbred piglets [HR = 0.20, 95% CI [0.05, 0.76], *p* = 0.018].

### 3.4. Piglet growth

**Figure 7:**
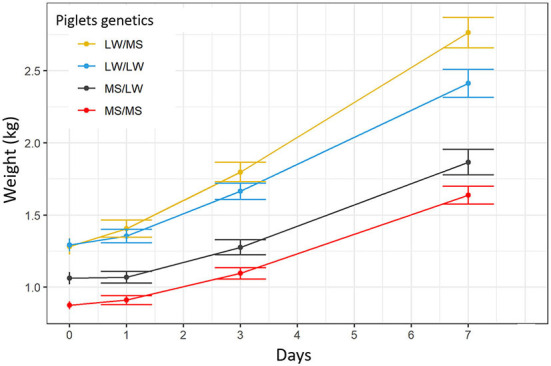
Growth curves for the four piglet genetic types (dam breed/sire breed).

The average birth weight was 875 ± 153, 1,063 ± 235, 1,292 ± 244, 1,283 ± 276 g for MS/MS, MS/LW, LW/LW, and LW/MS piglets, respectively. Birth weight differed significantly between pure piglets of the two breeds (*p* = 0.0003) and between pure MS and crossbred piglets born from LW sow (*p* = 0.001, Figure 7). The ADG over the D0 to D7 period was 110 ± 44 g/d for MS/MS piglets, 114 ± 56 g/d for MS/LW, 160 ± 63 g /d for LW/LW, and 208 ± 59 g/d for LW/MS piglets.

The results of the final linear model are given in Table 3. The three behavioral explanatory variables retained in the final model were PCAll on the period D-3 to D-1, PCStopNurse on D0 and PCStopNurse from D1 to D7. These three variables were considered in the model in two classes (S vs. M + I). The growth of piglets born from more restless sows before farrowing (PCAll, class S) was lower than that of piglets born from less restless sows (−22 ± 7 g/d, *p* < 0.0001). Growth was lower in piglets born from sows with more PCStopNurse on D0 (−45 ± 10 g/d, *p* < 0.001), and conversely, it was higher in piglets born from sows with a high PCStopNurse on D1–D7 (71 ± 9 g/d, *p* < 0.001). Litter size had a significant influence on piglet growth, the larger the litter, the lower the piglet growth (−24 ± 8 g/d between classes I and S). After correction for sow behavior, we still observed a significant influence of sow breed on piglet growth, with a lower growth for piglets from MS sow than piglets from LW sow (−103 ± 9 g/d).

**Table 3:** Estimates of the effects of behavioral traits and other fixed effects on ADG piglet growth (g/d) from D0 to D7.

	**Estimate**	**Standard error**	***T* value**	**Pr(<|t|)**
Intercept ^*^	229.50	11.46	20.03	< 0.0001
PCAll before farrowing (S)	−22.37	6.67	−3.35	< 0.0001
PCStopNurse on the day of farrowing (S)	−45.33	10.49	−4.32	< 0.0001
PCStopNurse after farrowing (S)	70.84	8.36	8.48	< 0.0001
Pen B	17.43	8.99	1.94	0.053
Pen C	−21.53	11.59	−1.86	0.065
Pen D	−14.65	10.41	−1.41	0.16
Mean litter size (M)	−24.18	9.64	−2.51	0.013
Mean litter size (S)	−23.67	8.01	−2.96	0.003
LW/LW	−38.42	7.65	−5.03	< 0.0001
MS/LW	−110.80	9.25	−11.98	< 0.0001
MS/MS	−102.55	9.28	−11.05	< 0.0001

* Estimates are expressed as the difference from the reference category. PCAll before (S) is the group of sows in which more numerous posture changes occurred and PCStopNurse (S) is the group of sows in which more numerous posture changes usable to end a nursing bout occurred.

## 4. Discussion

The evolution of EU animal welfare standards, with the End the Cage Age initiative (44), encourages researchers to expand their work on looser housing conditions for lactating sows. Identifying sow behavioral traits that are important for piglet survival and early growth when sows are kept in individual pens is essential to accompany the transition toward looser housing conditions to maintain production in maternal populations at a sustainable level. We have favorable indications that several behavioral traits determined by image analysis are usable to improve piglet survival in maternal lines. Indeed, direct genetic selection for piglet survival is not effective due to the low heritability of these traits [h^2^ = 0.05 on average; (45)]. Conversely, studies showed that sow behavior is moderately heritable [h^2^ = 0.10–0.40; (46)] and associated with piglet survival at the genetic level (47, 48).

The two breeds included in this study have different ranges in reactivity (46) and markedly differ in speed of movement (23, 25). Using breeds with different behavioral reactivity to estimate relationships between sow behavior and piglet survival is an insightful approach to explore the diversity of cases that exist in a large population. Our aim was to demonstrate that beyond the discrepancies, the individual behavior of a sow rather than the breed to which she belongs has a major role in piglet survival (1, 17, 49). Variations in behavior were observed within each breed and exceeded variation between breeds so that sows from the two breeds were often grouped in the same cluster. Variations among sows in a population can be exploited to improve performance, especially piglet survival under looser housing conditions.

For this study, sows of both breeds were reared together in the same farrowing unit. They were kept in large individual pens, accustomed to humans and visited several times a day. Their environment was enriched (50) and they should have experienced less stress than more constrained animals (51, 52). Accordingly, stillbirth was very low (3.8%) and the survival rate of liveborn piglets was 80% in LW, which is higher than what was reported by other researchers under similar housing conditions (75%) (6, 53). However, we observed effects of the micro-environment on performance, e.g., the location of the pen in the room. Sows located closer to the central corridor where the litters were weighed each day had a lower piglet survival. Accordingly, Lensink et al. (54) estimated a positive association between sow reactivity to piglet handling for routine management with piglet crushing. Sows located nearby were more disturbed than other sows and at greater risk to develop a reaction toward screaming piglets (55), even more so if they were in first parity (56). Conclusions drawn from our study were formulated after correction for this effect. Performance is also a result of interactions between the sow and her offspring. The activity patterns of the 21 LW and 22 MS sows were analyzed jointly. Due to heterosis effects, crossbred piglets show higher vigor than purebred piglets (57–59). In order to focus the study on sow potential for piglet survival and growth, the genetic type of the piglet was systematically corrected for in the piglet analyses.

For a sow's farrowing to be successful, the restlessness associated with its preparation must gradually give way to calm. Behavioral patterns reveal more about sow maternal abilities than punctual observations. Our study relied on the use of image analysis by convolutional neural networks to predict sow behavior in the long term. This approach made it possible to quantify behavior that describes sow activity, considering postural activity during the time spent daily in different postures, postural changes, and, specifically, standing activity that included being in motion or not, and exploring or not the environment (rooting, pawing, etc.), in addition to maintenance activities (drinking or eating).

The use of postural and activity budgets is rarely described in the literature [however, see (60–63)]. There are many studies of postural changes assessed by scan sampling and rare continuous analyses of behavior over more than 1 day. The effect of activity budget on performance was investigated in relation to posture (17, 62), nest building, and dam-piglet interaction (15). Considering activity as compositional data was advantageous to combine complementary sources of information in a single analysis. Over the before-farrowing period and the after-farrowing period, groups of sows with different activity patterns were identified, and some of the differences between groups were essential for piglet survival or growth. Studies of larger populations will most certainly highlight the contrasts between these different sow profiles (groups) once again.

Sows naturally become more restless in the run-up to farrowing, which is manifested in particular by a higher proportion of time spent standing. Sows from the current study spent 35% of their postural budget standing in the 24-h period before farrowing, in line with Rosvold et al. (63) who reported 40% in the 12 h before farrowing, and a third of this time was spent nest-building. This activity should be more elaborate and less fragmented than in a crate (64). It is frequent in sows that receive straw daily (1) and can last 15 h (65), thus accounting for a large proportion of the standing-activity budget. In our study, sows that spend more of their standing time doing something other than eating and drinking had litters with a higher survival rate. Consistently, it is mentioned in the literature that nest-building activity, especially when performed satisfactorily for behavioral needs, is associated with fewer piglets crushed (9, 13, 16, 66) and starved to death (67).

It is possible that sows that are standing and performing activities other than eating and drinking before farrowing are actively preparing for farrowing. Later, these sows sniff their piglets more than others (63). Videos are available to confirm that fine phenotypes (manipulating straw, sniffing piglets before lying) correlate with the time spent standing, for example. The timing of nest-building activity is a key parameter for farrowing success. It would be wise to analyze the time interval between the two activities with advanced image analysis. In addition, if the key activity of nest-building is impaired, it results in prolonged farrowing (68) and has long-term consequences, such as sows changing posture more frequently in the 24 h after farrowing (69, 70). We found a strong association of before-and-after sow activity (r*p* = 0.70). There are therefore associations between different or the same behaviors performed in separate time windows. Prepartum activity can predict sow behavior after parturition, as discussed in (9, 65, 70, 71). We found a pronounced relationship with piglet survival: a piglet had a 44% lower instantaneous risk of dying if it was born from a sow that was more restless prepartum (PCAll). We also found that the most restless sows before farrowing (PCAll) produced piglets with reduced daily growth until D7. To our knowledge, the effect of restlessness before farrowing on piglet growth was not quantified previously. Nonetheless, and although not confirmed by Ocepek et al. (13), it was found that sows with active nest-building prepartum had a better nursing performance, which resulted in higher piglet weight gain in early lactation (72).

On the day of farrowing, one cluster encompassed sows that were more restless, presumably because of a delay in the nest-building activity or because they immediately interacted with the newborn piglets. This shift of part of the activity expected from before farrowing to the farrowing period is likely to occur more often in primiparous sows (17). As regards the standing-activity budget, one cluster was characterized by a higher proportion of time spent drinking, an activity known as important for piglet survival (73). However, we did not find any influence of either sow postural budget or standing activity or postural changes on piglet survival in this period, which disagrees with several studies (8, 17) [review in (20)]. Consequently, we did not verify the classical assumption that piglet survival in this period is linked to postural changes that are risky for the piglets. Since the piglets spend time in proximity to the sow earlier in the period (19), the probability of survival is lower in sows that roll on the side (20, 36) and that use the sitting-lying transition more often. Yet, Weary et al. (38) and Damm et al. (20) did not find any effect of the frequency of postural changes on piglet survival the day of farrowing. We did not test the interaction of postural changes with breed due to the small size of our dataset. It could be relevant if the two sow genotypes have different abilities to control body movements (36), which is assumed, considering the slow motion of MS sows. The daily weight gain of each piglet was 45 g lower in sows that were more restless, i.e., that used more postural changes to presumably limit access to the udder than the others. We assumed that in restless sows, colostrum intake, which greatly determines early growth (74, 75), is lower, since we know from the literature that they tend to be less involved in the nursing activity (3, 39). Muns et al. (76) showed that piglets born from stressed sows have a lower daily weight gain during lactation than piglets born from unstressed sows. Activity is only a partial indicator of stress (77), so we cannot draw immediate conclusions for our animals.

In the period from D1 to D7 in early lactation, in the continuity with D0, on average patterns of activity did not change. Sows spent 90% of their time lying (12). When their udder is exposed, sows provide a warm and comforting environment for the piglets (12, 78) to suckle and rest (19, 79). It is also important that sows progressively resume activity (11), which is punctuated by nursing bouts (3, 39). Yet, time spent lying remained stable until D7. As for this aspect, we found that the groups differed in the time spent exposing their udder while lying, which has an effect on the lying-activity budget. As regards the standing-activity budget, the two groups differed, with one group more involved in activities other than maintenance. Presumably, those sows interacted with the physical environment (rooting) and piglets more than the ones from the two other groups. We found that piglet survival was higher in sows that spent more time lying with their udder exposed during the D1–D7 period. Calm sows promote piglet survival. The other components of the postural budget did not explain piglet performance. Only a few studies have attempted to draw such conclusions [sitting time with crushing; (80)]. We also found that sow restlessness until D7 had a positive influence on piglet survival. Such a finding is counter-intuitive and in contradiction with, e.g., (9, 12, 81, 82), who all reported that less active sows crush fewer piglets. Perhaps in our population, the more restless sows were the ones that bonded best with their piglets and that were therefore careful not to crush them, in agreement with (12, 78). We and other researchers did not confirm that postural changes like standing-to-lying are unfavorable to piglet survival (36, 38). According to (14), more frequent posture changes correlate with repeated missing of milk ejection and lead to lower piglet survival. We interpreted certain postural changes related to the interruption of nursing bouts. In particular, rolling limits piglet access to the udder (83) and, consequently, could result in lower piglet growth (3, 6, 84). Yet, counter-intuitively once again, we found that daily piglet growth was 71 g greater in sows that hide the udder more often than the others. The explanation could be that sows respond by changing their posture for piglets that are more eager to suckle, which are those with greater growth. The reason for the sow to change posture can also be a response to signals from weak piglets that do not have access to milk (37, 85). The activity of the piglets at the udder is influenced, in turn, by the activity pattern of the sows (86, 87). Sows that spend more time lying with the udder exposed are likely to produce calmer piglets with a synchronized suckling activity and that may fall asleep more frequently at the udder. Conversely, sows that raise piglets that compete at the udder share fewer resting periods in contact with their piglets (36). By reacting, they might promote survival of starving and weak piglets, with the next intake less distant in time than one would expect from an unresponsive sow (67).

There is a genetic root to the reactivity of pigs (46, 88). Even newborns that are naïve could behave like their dam—more reactive sows, more reactive piglets—and we could assume that piglets could thereby be more able to avoid being crushed by their dam. Sows that interrupt contact of their piglets with the udder encourage them to spend more time in the creep area, where they gain more weight (86). As mentioned above, maybe sows that change postures more often have more interactions with their piglets (2, 9), but the association of dam-piglet interactions with survival and growth is poorly known. It would be useful to analyze the suckling activity of the piglets with AI, consistent with the image-based work in previous studies (89, 90). This would help us to understand whether changes in sow posture depend on solicitation by the piglets.

The ethological interpretation of the results is constrained by the fact that we only analyzed the postures and standing-activity patterns of the sows. It would be opportune to use scan sampling or continuous analyses to verify the relationships between the behavioral traits predicted from the algorithm and maternal behavioral traits (nest-building before farrowing, nose contacts after farrowing) to better understand their influence on piglet performance. If future technological developments allow for the analysis of fine-grained behavioral traits, contrasts between the two breeds might surface according to straw manipulation and the frequency of nose contacts with piglets, in favor of the MS (25, 27). Han et al. (91) found that sows crossed with a Chinese line made more postural changes than those crossed with a white line but nevertheless had higher piglet survival. Here and in a previous study, we did not find any difference between MS and LW sows in the frequency of posture changes (27). Certain sows appear to be more attentive, careful, and protective when changing posture than others (15, 70), and this favors piglet survival. Speed of movement could be an important trait that explains why MS sows crush fewer piglets [e.g., (92, 93)]. It was not reasonable to estimate this trait, given our level of confusion about these postures, but it would be possible with a more efficient algorithm. Better prediction of activities might be achieved with the use of a more recent version of Yolo (94).

We found that sow restlessness is a repeated trait on the days around farrowing, with a peak at D-1. Consistently, Harris and Gonyou (95) found that crated gilts change posture in the before-farrowing period four times more often than in the after-farrowing period. Furthermore, Thodberg et al. (96) observed that gilts active on the day of farrowing continue to be so on the first day after farrowing. Sows that perform more nest-building activity before farrowing also have an activity more orientated toward the piglets during early lactation (63). Restlessness before farrowing could describe the sow behavioral profile and could be used for breeding if it refers to a repeatable trait over parities. Vangen et al. (48), using an on-farm notation from 1 to 7, estimated that sow interruption and nervousness while nursing is a heritable trait (h^2^ = 0.08). Stratz et al. (97) reported that it is genetically correlated with the behavior on the day of farrowing (r*g* = 0.55) and with low piglet performance because the index they used also described sows that were aggressive toward their piglets. In their situation which included lowly maternal sows, sows that stood more frequently during the farrowing process limited piglet milk intake. Selecting sows that are not too extreme for the behavior of interest is recommended. On the basis of our findings, we conclude that the elimination of sows that are too calm outside the farrowing period appears to be a relevant solution as well. For any breeding strategy, an optimum should be targeted to avoid trends unacceptable with respect to animal welfare. Selection designed on the basis of the use of automated longitudinal behavioral analysis should be preferable to selection based on behavioral notations from humans. This would ensure the acquisition of standardized and high-throughput measures from which criteria that effectively reflect the behavioral profile of each sow could be developed.

## 5. Conclusion

A wide range of variations underpinned the activity patterns of the two breeds. The longitudinal study highlighted some coherent and some different effects of sow activity on piglet performance over the three periods around farrowing. The live-born piglets of sows that were more active the day before farrowing, standing and in other activity than drinking and eating, had higher survival rates. Sows that were more restless before farrowing remained so in early lactation, and this attitude had a negative effect on piglet growth and a positive effect on piglet survival, respectively, in each period. Specifically, the growth of piglets of sows that changed posture more often on the day of farrowing was reduced, whereas such behavior, if performed later, favored piglet growth. Several sow activities that occur in different time windows explain piglet survival and growth. Breeding for sow behavioral criteria with the support of AI might be a relevant strategy to improve sow and piglet performance.

## Data availability statement

The raw data supporting the conclusions of this article will be made available by the authors, without undue reservation.

## Ethics statement

The experimental protocol was designed in compliance with Legislations of the European Union (Directive 86/609/EEC) and France (Decree 2001–464 29/05/01) for the care and use of animals (Agreement For Animal Housing Number C-35-275-32).

## Author contributions

LC conceived the project. LC, YB, and JB carried out the experiment. LC and JB collected data on the farm. OG, LC, ID, and MB participated in the data analysis and wrote the manuscript. All authors contributed to the article and approved the submitted version.
